# -174G>C interleukin-6 gene polymorphism in Tunisian patients with coronary artery disease

**DOI:** 10.4103/0256-4947.75777

**Published:** 2011

**Authors:** Lakhdar Ghazouani, Nesrine Abboud, Sonia Ben Hadj Khalifa, Faouzi Added, Ali Ben Khalfallah, Brahim Nsiri, Mounira Mediouni, Touhami Mahjoub

**Affiliations:** From the a Research Unit of Biology and Genetics of Cancer, Haematological and Autoimmune Diseases, Faculty of Pharmacy of Monastir, Monastir, Tunisia; b Fattouma Bourguiba Hospital Center, Monastir, Tunisia; c Menzel Bourguiba Hospital Center, Bizerte, Tunisia; d Hospital Center of Jendoubad, Tunisia

## Abstract

**BACKGROUND AND OBJECTIVES:**

A state of low-grade inflammation accompanies the pathogenesis of atherosclerotic events. Interleukin-6 (IL-6) is a pleotropic pro-inflammatory cytokine that modulates the development of acute coronary syndromes (ACSs), partly by destabilizing coronary atherosclerotic plaques. We have examined the contribution of the -174G>C IL-6 promoter variant on the risk of coronary artery disease (CAD) among Tunisians.

**PATIENTS AND METHODS:**

Study subjects included 418 CAD patients and 406 age- and sex-matched controls. IL-6 genotyping was done by PCR–restriction fragment length polymorphism.

**RESULTS:**

The frequency of the -174C allele (mutant) was lower in Tunisians than in Europeans, and the distribution of -174 G>C genotypes was similar between CAD patients and control subjects. Moreover, compared to GG genotype carriers, -174C allele carriage did not increase the CAD relative risk (odds ratio and 95% confidence interval=1.09 and 0.80-1.49), which remained nonsignificant after adjusting for traditional risk factors for CAD (age, smoking, hypertension, diabetes and obesity).

**CONCLUSION:**

The -174G>C IL-6 promoter variant is not associated with an increased risk of CAD among Tunisians.

Coronary artery disease (CAD) is a major cause of death and disability in developed countries, and is characterized by a long asymptomatic developmental phase, which progresses to precipitation of atherosclerotic plaques, the fatty streak being the earliest visible lesion.^1^ Atherosclerosis is regarded as a state of chronic low-grade inflammation occurring within the arterial wall,^2^,^3^ linked with local recruitment of neutrophils and monocytes, and the presence of activated macrophages in the cap of the atherosclerotic plaque,^4^ which in turn contributes to plaque rupture by affecting matrix metalloproteinases. Pro-inflammatory cytokines contribute to the onset and/or to the progression of atherosclerotic manifestations, through destabilizing coronary atherosclerotic plaque, and induction of acute coronary syndromes (ACSs).

Interleukin-6 (IL-6) is a 26-kDa pleotropic inflammatory cytokine produced by many cell types, including fibroblasts, monocytes, adipocytes and endothelial cells^5^,^6^ and is reportedly associated with endothelial damage and initiation of atherosclerotic events.^5^,^7^ IL-6 belongs to the cytokine family, which includes IL-6, IL-11, ciliary neurotrophic factor, cardiotrophin-1 (CT-1), cardiotrophin-like cytokine, leukemia inhibitory factor, neuropoietin and oncostatin M,^8^,^9^ together with IL-27 and IL-31.^10^ Mechanistically, IL-6 contributes to CAD development by affecting metabolic, endothelial and coagulant events, and is viewed as a local and circulating marker of coronary plaque inflammation. IL-6 was implicated in the pathogenesis of ischemic cardiovascular events, including unstable angina^11^ and ACS,^12^ and its expression and secretion are regulated by IL-1 and TNF-α, which are highly induced in the atherosclerotic plaque.^13^,^14^ IL-6 induces the expression of tissue factor, monocyte chemotactic protein-1, matrix-degrading enzyme and low-density lipoprotein receptors in macrophages, and stimulates the aggregation of platelets, proliferation of vascular smooth muscle cells and production of C-reactive protein and fibrinogen.^13^,^15^

Polymorphic variants in the IL-6 gene were implicated in the expression of acute-phase proteins and were described as regulators of atherogenic markers. Of these, the commonly studied polymorphism is the functional variant -174G>C. Association of this single nucleotide polymorphism (SNP) with CAD has been reported in some but not all the studies.^16^–^19^ We investigated the association of -174G>C IL-6 promoter variant with CAD risk, after controlling for traditional CAD risk factors (age, obesity, hypertension, diabetes mellitus and smoking).

## PATIENTS AND METHODS

This was a case-control study involving 418 CAD patients (87 females and 331 males) diagnosed as having CAD based on criteria given by the World Health Organization (WHO), and who attended the Farhat Hached Hospital in Sousse, Tunisia. Diagnosis of CAD was based on coronary angiography (obstructive coronary lesions with >50% narrowing of any subepicardial coronary artery). Determination of CAD severity was based on the number of major epicardial coronary arteries affected. Exclusion criteria included concomitant renal, hepatic or autoimmune disease. Obesity was defined as body mass index (BMI) of 30 kg/m^2^ or higher. Hypertension was defined as blood pressure (BP, right arm) >140/90 mm Hg on two separate occasions, measured using mercury sphygmomanometer with participants in the sitting position following a 5-minute period of rest (the mean of two readings measured 1 minute apart was adopted) and/ or the use of antihypertensive therapy (ACE inhibitors, angiotensin receptor blockers, beta blockers, diuretics and calcium antagonists). Diabetes was diagnosed according to WHO criteria (fasting blood glucose >12.6 mg/dL or 70 mmol/L) on at least two occasions and/or the use of glucose-lowering drugs, including insulin).

The control population was comprised of 406 healthy subjects (107 females and 299 males) who either attended a routine health check at a general practice or at their workplace. They were required to have a normal physical examination, a normal resting electrocardiogram and no personal or family history of CAD. All participants were asked to fill a standard questionnaire detailing demographic details, together with data on traditional CAD risk factors. All subjects were asked to sign a written consent form after the aims and details of the study were explained to them. The study was approved by the Ethics Committees of the University Hospital of Sousse, Tunisia.

Blood for IL-6 genotyping was collected in EDTA tubes and kept frozen pending DNA extraction. Total genomic DNA was extracted from the buffy coat layer by the salting-out procedure and was stored in TE buffer (10 mmol/L Tris-HCl, 1 mmol/L EDTA, pH 8.0). Genotyping of -174G>C (Nla III) was performed by PCR-restriction fragment length polymorphism (PCR-RFLP) as described.^20^ The following primers were used to amplify a 525-bp fragment containing -174 sites: 5’-GGA GTC ACA CAC TCC ACC T-3’ (sense) and 5’-CTG ATT GGA AAC CTT ATT AAG-3’ (antisense). Digested fragments were separated by electrophoresis on 3% ethidium bromide-staining agarose gels and were visualized by UV transillumination.

Statistical analysis was carried out using SPSS version 13.0 software (SPSS, Chicago, IL). Data were expressed as mean (standard deviation) for continuous variables or as percentages of the total for categorical variables. The *t* test was used for analyzing continuous variables (age, BMI, plasma lipid levels), and chi-square analysis was employed for analyzing categorical variables (gender, hypertension, smoking, diabetes); a *P* value <.05 was considered statistically significant. The associations of specific genotypes with CAD were determined by logistic regression analysis, adjusted for age, smoking, hypertension, diabetes and body mass index (BMI). Taking homozygous carriers of the wild-type allele as reference, two independent odds ratios (ORs) were estimated, one for heterozygote carriers and the other for homozygote carriers.

## RESULTS

**Table 1** lists the demographic and clinical characteristics of the 418 CAD patients and 406 control subjects. The two groups were matched for gender (*P*=.06) and age (*P*=.11). CAD patients had a higher BMI (*P*<.001), systolic (*P*<.001) and diastolic (*P*=.006) blood pressure, and higher total cholesterol (*P*<.001), triglyceride (*P*<.001), creatinine (*P*<.001) and urea (*P*<.001) levels and lower HDL-cholesterol levels (*P*<.001). The prevalence of hypertension (*P*<.001) and rate of smoking (*P*<.001) were higher among patients than among control subjects.

**Table 1:** Demographic and clinical characteristics of study subjects.

Characteristics	Controls (n=406)	Patients (n=418)	*P*
Females: n (%)	107 (26.4)	87 (20.8)	.071
Mean age (years)	56.7 (14.12)	58.1 (12.0)	.092
Mean BMI (kg/m^2^)	25.22 (2.35)	27.08 (4.20)	< .001
Hypertension: n (% total)	54 (13.3)	134 (32.1)	< .001
Systolic BP (mm Hg)	120.0 (13.7)	133.8 (26.4)	<.001
Diastolic BP (mm Hg)	73.7 (8.9)	76.2 (13.7)	.006
Fasting glucose (mmol/L)	5.7 (1.7)	9.5 (5.3)	<.001
Diabetes: n (% total)	45 (11.1)	198 (47.4)	<.001
Smoking: n (% total)	104 (27.4)	234 (56.8)	<.001
Urea (mmol/L)	4.64 (1.86)	6.77 (4.31)	<.001
Uric acid (mmol/L)	301 (106)	292 (119)	.59
Creatinine (mmol/L)	75 (16)	104 (72)	<.001
Total cholesterol (mmol/L)	3.98 (1.24)	4.78 (1.22)	<.001
Triglycerides (mmol/L)	1.35 (0.86)	1.76 (0.95)	<.001
HDL-cholesterol (mmol/L)	1.30 (0.44)	1.07 (0.45)	<.001
LDL-cholesterol (mmol/L)	2.27 (1.19)	3.00 (1.09)	<.001

Values are number (percentage) or mean (standard deviation).

### 

#### Genotype and allele frequencies

Genotype distribution of the -174G>C polymorphism did not deviate from the Hardy-Weinberg equilibrium among participants (**Table 2**). No statistically significant differences in genotypes or allele distribution were seen between patients and controls, and -174 G>C did not exhibit transmission distortion. The distribution of the minor allele -174C (15.5% vs. 14.3%), and -174G/C (26.3% vs. 25.1%) and 174C/C (2.4% vs. 1.7%) genotypes was comparable between patients and controls, respectively. An increased prevalence of -174C allele was seen among patients with unstable angina (16.1%) or myocardial infarction (17.0%) patients compared to stable angina (8.6%) patients (**Table 3**).

**Table 2:** IL-6 genotype and allele frequencies.

-174G>C IL-6 genotypes	Controls (n=406)	CAD patients (n=418)
GG	297 (73.2)	298 (71.3)
GC	102 (25.1)	110 (26.3)
CC	07 (1.7)	10 (2.4)
IL-6 alleles		
G	696 (85.7)	706 (84.5)
C	116 (14.3)	130 (15.5)

Value are number (percentage).

**Table 3:** Genotype and allele distribution according to CAD severity.

-174G>C IL-6 Genotypes	Myocardial infarction (n=215)	Unstable angina (n=112)	Stable angina (n=29)
GG	147 (68.4) 2	79 (70.5)	24 (82.8)
GC	63 (29.3)	30 (26.8)	05 (17.2)
CC	5 (2.3)	03 (2.7)	00 (00)
IL-6 alleles			
G	357 (83.0)	188 (83.9)	53 (91.4)
C	73 (17.0)	36 (16.1)	05 (8.6)

Value are number (percentage).

#### Regression analysis

The association of -174G>C IL-6 variant with CAD was examined first by univariate regression analysis and later by multivariate regression analysis. Taking -174G/G genotype as the reference, the univariate regression analysis demonstrated no significant association of -174G/C genotype (OR=1.07; 95% CI=0.78-1.47) or -174C/C genotype (OR=1.42; 95% CI=0.53-3.79) with CAD. Multivariate regression analysis confirmed the lack of association of -174G/C genotype (OR= 1.21; 95% CI= 0.74-1.97) or -174C/C genotype (OR=0.89; 95% CI=0.19-4.02) with CAD, after adjusting for CAD traditional risk factors (age, smoking, BMI, hypertension and diabetes mellitus) (**Table 4**). This lack of association was irrespective of whether a dominant or recessive effect of the allele was assumed.

**Table 4:** Unadjusted and adjusted odds ratios for CAD.

IL-6 Genotypes	Unadjusted OR (95% CI)	*P*	Adjusted OR^a^ (95% CI)	*P*
GG	1.00 (Reference)		1.00 (Reference)	
GC	1.07 (0.78-1.47)	.65	1.21 (0.74-1.97)	.43
CC	1.42 (0.53-3.79)	.47	0. 89 (0.19-4.02)	.89
GG/GC vs. CC	1.39 (0.52-3.71)	.52	0.82 (0.19-3.56)	.79
GG vs. GC/CC	1. 09 (0.80-1.49)	.55	1.17 (0.73-1.89)	.49

a Adjusted for age, smoking, hypertension, diabetes and body mass index.

## DISCUSSION

Atherosclerosis, inflammation and thrombosis are hallmarks of ACSs.^1^,^13^,^21^ It is well established that inflammatory processes are key factors in the pathogenesis of atherogenesis and ACSs.^2^,^21^ IL-6 is a pleiotropic pro-inflammatory cytokine and a key mediator of inflammation in both low-grade asymptomatic inflammation within the atherosclerotic plaque and the acute reaction of a thrombotic coronary event.^22^,^23^ This was highlighted by findings demonstrating that elevated IL-6 levels predict the onset of future coronary events in apparently healthy individuals, as well as mortality in ACS patients.^22^,^24^

To the best of our knowledge, this is the first report describing the association of IL-6 promoter polymorphism -174 G>C with CAD in Tunisians. The main finding in this study was the lack of association of -174G>C alleles or genotypes with CAD. The frequency of the -174C allele was comparable between CAD patients and healthy controls, but was lower than that in European populations. In addition, the frequency of the -174G/C (26.3) and -174C/C (2.4) genotypes in our patient group was lower than that in French or Irish (Belfast) population, as reported in the Etude Cas Temoin de l’Infarctus du Myocarde (ECTIM) study,^25^ or in German or Scottish populations.^19^,^26^ Our finding of an apparent lack of association of -174G>C alleles and genotypes with CAD was reminiscent of an earlier case-control study among Tunisian patients of breast carcinoma (immuno-inflammatory disease) (n=305) and healthy control subjects (n=200), where a comparable distribution and frequency of -174G>C alleles and genotypes were noted between patient and control groups,^27^ which were also comparable to those reported by Sekuri et al in the Turkish population.^28^

Regression analysis confirmed the lack of association of -174G/C genotypes with CAD. Our results are in accord with an earlier study demonstrating the lack of association of the G/C genotype, and a weak but statistically nonsignificant association of the C/C genotype, with increased CAD risk^19^; and with the ECTIM study, where comparable CAD risk estimates for -174G/C and -174C/C genotypes were reported.^25^ In contrast, the Northwick Park Heart Study, a prospective study of 2751 middle-aged healthy men, reported that the risk imparted by the -174G/C genotype was higher than that imparted by the -174C/C genotype.^17^ The same study demonstrated a weak but significant association of the -174G>C variant with increased CAD risk, especially among smokers -174G/C genotype carriers. A recent Greek study with a limited number of patients (26 with stable angina, 45 with unstable angina and 58 with nonfatal myocardial infarction) demonstrated enrichment of the -174^*^C allele in patients with myocardial infarction (compared with patients with stable and unstable angina), suggesting that the -174G/C variant may be involved in CAD pathogenesis.^29^

Given the well-established role of inflammatory mechanisms in CAD pathogenesis, coupled with the role of IL-6 as a pro-inflammatory cytokine, it is tempting to speculate that induction of inflammation mediated by altered IL-6 levels regulated by specific IL-6 variants may modulate CAD development and progression. Taken together, these findings suggest that the carriage of the -174C allele imparts a weak CAD association, compared to the -174G/G genotype carriers. Our findings suggest that genetic variations in the IL-6 gene are unlikely to play an important role in the genetic predisposition to CAD, and that it represents a poor risk indicator in patients with CAD.

The strengths of this study lie in the sample size used (418 patients and 406 control subjects), which was sufficiently powered to reduce type I errors; and in being the first to examine the association of IL-6 promoter variants with CAD in the ethnically homogenous Tunisian population. However, our study has some limitations; namely, that we were not able to measure plasma IL-6 concentrations in patients and controls, and that it was limited to a specific ethnic group (north African Tunisian Arabs), thereby necessitating follow-up studies in CAD patients from different ethnic groups. Another limitation of the study is that IL-6 genotyping was determined in confirmed CAD patients, thus questioning the value of analyzing these variants, as it is not clear whether mutation-carrying control subjects may develop future CAD events. The possible involvement of other IL-6 variants, as well as nearby or distant functional gene polymorphisms, in disease pathogenesis remains to be seen. Our study is of potential interest since comparison of genetic associations among different populations with possibly different SNP frequencies is useful.
